# Optimization of microbial fuel cell performance application to high sulfide industrial wastewater treatment by modulating microbial function

**DOI:** 10.1371/journal.pone.0305673

**Published:** 2024-06-18

**Authors:** Nattawet Sriwichai, Rutrawee Sangcharoen, Treenut Saithong, David Simpson, Igor Goryanin, Nimaradee Boonapatcharoen, Saowalak Kalapanulak, Pornpan Panichnumsin

**Affiliations:** 1 Center for Agricultural Systems Biology, Pilot Plant Development and Training Institute, King Mongkut’s University of Technology Thonburi (Bang Khun Thian), Bangkok, Thailand; 2 Pilot Plant Development and Training Institute, King Mongkut’s University of Technology Thonburi (Bang Khun Thian), Bangkok, Thailand; 3 Bioinformatics and Systems Biology Program, School of Bioresources and Technology, King Mongkut’s University of Technology Thonburi (Bang Khun Thian), Bangkok, Thailand; 4 Biological Systems Unit, Okinawa Institute of Science and Technology, Okinawa, Japan; 5 Excellent Center of Waste Utilization and Management, National Center for Genetic Engineering and Biotechnology, National Sciences and Technology Development Agency at King Mongkut’s University of Technology Thonburi, Bangkok, Thailand; Nanjing University of Science and Technology, CHINA

## Abstract

Microbial fuel cells (MFCs) are innovative eco-friendly technologies that advance a circular economy by enabling the conversion of both organic and inorganic substances in wastewater to electricity. While conceptually promising, there are lingering questions regarding the performance and stability of MFCs in real industrial settings. To address this research gap, we investigated the influence of specific operational settings, regarding the hydraulic retention time (HRT) and organic loading rate (OLR) on the performance of MFCs used for treating sulfide-rich wastewater from a canned pineapple factory. Experiments were performed at varying hydraulic retention times (2 days and 4 days) during both low and high seasonal production. Through optimization, we achieved a current density generation of 47±15 mA/m^2^, a COD removal efficiency of 91±9%, and a sulfide removal efficiency of 86±10%. Microbiome analysis revealed improved MFC performance when there was a substantial presence of electrogenic bacteria, sulfide-oxidizing bacteria, and methanotrophs, alongside a reduced abundance of sulfate-reducing bacteria and methanogens. In conclusion, we recommend the following operational guidelines for applying MFCs in industrial wastewater treatment: (i) Careful selection of the microbial inoculum, as this step significantly influences the composition of the MFC microbial community and its overall performance. (ii) Initiating MFC operation with an appropriate OLR is essential. This helps in establishing an effective and adaptable microbial community within the MFCs, which can be beneficial when facing variations in OLR due to seasonal production changes. (iii) Identifying and maintaining MFC-supporting microbes, including those identified in this study, should be a priority. Keeping these microbes as an integral part of the system’s microbial composition throughout the operation enhances and stabilizes MFC performance.

## Introduction

The circular economy model is gaining traction as a promising approach to tackle the pressing global problem of resource overuse. This approach is primarily driven by the idea of achieving “zero-waste,” which emphasizes the importance of reusing and recycling existing materials and products. In the wake of growing awareness of the implications of global climate change and unsustainable resource utilization, research in waste management and the advancement of alternative, environmentally friendly, and renewable energy sources have become a global challenge [1]. Microbial fuel cells (MFCs) have emerged as a promising technology for the realization of resource efficiency, waste reduction and sustainability.

The MFC technology, by the niche of microbial activity, can remove organic and inorganic contained in wastewater and simultaneously produce electricity [2]. MFCs typically require a short start-up time, and the generation of electrical power can reach a steady state within a few days of operation [3]. MFCs can function effectively at low temperatures and low organic waste levels, under which other wastewater treatment methods tend to struggle [4]. Moreover, MFCs are well-suited for removing toxic contaminants such as ammonia, nitrate, sulfur, and iron. They can effectively convert carbon and sulfur wastes to generate electricity [5, 6]. While MFCs have demonstrated potential for improving the quality of a broad range of wastewater, including household, winery, and food processing wastewater, their practical use in industrial wastewater treatment is still limited [2, 7].

Under industrial settings, anaerobic digestion (AD) is often preferred for wastewater treatment, due to its greater practicality. While the AD system can effectively reduce COD, the effluent often retains a repulsive odor and a dark color due to the remaining sulfide residues. Consequently, further treatment is required, such as the use of an aeration pond [8]. The integration of MFCs into the AD process represents an innovative treatment method with the potential to usher in a new generation of globally conscious agro-industry. Compared to conventional post-AD treatment methods like activated sludge, stationary ponds, and wetlands, MFCs represent a more sustainable and efficient treatment system in terms of energy recovery, efficiency, and the environmental impact [9, 10]. MFCs can serve as a polishing step by effectively removing both organic and inorganic compounds that may remain in the effluent. Additionally, MFCs can reduce odors since their electrochemical reactions can help mitigate odor issues associated with the AD process, reducing the release of odorous gases [9]. MFCs have a relatively compact design and can be easily integrated into existing treatment systems or retrofitted into limited spaces. MFCs typically generate less excess sludge, which could reduce energy costs linked to sludge disposal by 45–75% compared to aeration-based treatments [10, 11]. MFCs can potentially lower the need for chemical usage to meet effluent quality standards, unlike the activated sludge process, which requires the addition of chemical compounds, resulting in high running costs and the introduction of contaminants into the water [12].

Factors influencing MFC operation have been extensively studied, with a primary focus on COD removal and current density generation. The most-studied factors related to operating conditions include the types and composition of organic substrates, organic loading rate (OLR) and hydraulic retention time (HRT) [11]. During long-term operation, MFCs are exposed to changing wastewater characteristics, which can result in alterations in the OLRs within the operating reactor and disrupt the equilibrium of electrical production. The effect was shown to be mitigated by adjusting the HRT, a key determinant of MFC operation [13]. HRT modulates MFC equilibrium by controlling both the amount of organic loading to the reactor and the duration for which the organic compounds are exposed to MFC microorganisms. It was shown that a decrease in HRT resulted in a reduction in the maximum voltage generated by a dual-chamber MFC for treating municipal wastewater [11, 14]. Variations in the operating conditions can impact the composition and activity of the microbial community in MFC, which crucially determine the overall performance of the system [15–17].

Microorganisms in the MFC reactor function as biocatalysts for anaerobic digestion in the anodic chamber. By facilitating the conversion of substrates into electricity and the reduction in COD, they directly influence the overall performance of MFCs [18]. The microbial community in an MFC system comprises diverse functional groups that work synergistically to facilitate the conversion of organic compounds to electricity [19]. In the literature, widely reported MFC microbes include the genera *Pseudomonas–*which can utilize organic compounds for hydrogen production and electricity generation by electron transfer to anodes [20], and *Zoogloea–*which can oxidize organic compounds like sugar or alcohol and is involved in electricity generation [21]. Exoelectrogenic bacteria (EB) such as *Arcobacter*, *Geobacter*, *Dysgonomonas*, and *Desulfovibrio* are also involved in power generation [16]. The performance of MFCs in treating complex wastewater is dependent on interactions among several microbial groups within the anodic chamber [19]. Besides EB, dominant microbes in MFC systems include hydrolytic/fermentative bacteria, which readily provide substrates for the EB. Examples of these functional genera include *Azoarcus* and *Comamonas*, which are involved in the digestion of complex compounds; *Plasticumulans*, responsible the digestion of acetic and small fatty acids; and *Methylomonas*, which assists in methane oxidation and electricity generation [15]. Conversely, the genus *Methanosaeta* utilizes acetate and produces methane as an end-product, causing limited free-electron transfer to the anode and, consequently, lowering electricity output [17]. Therefore, maintaining a balance among these microbial groups and their expressed functions is essential in determining MFC performance.

To facilitate practical, real-world applications, this research aimed to enhance the effectiveness and suitability of MFCs for treating sulfide-rich wastewater from the canned pineapple industry. We investigated the effects of process optimization and variations in the hydraulic retention time (HRT) and organic loading rate (OLR) on microbial communities within MFC systems. The optimized MFC system exhibited impressive performance metrics, namely a current density of up to 88 mA/m^2^, a COD removal efficiency of 99%, and a sulfide removal of 97%. Moreover, practical guidelines for industrial-scale application of MFCs for wastewater treatment were proposed. Also, we identified specific microbial communities that support MFC performance.

## Materials and methods

### MFC configuration and wastewater properties

**Fig 1:**
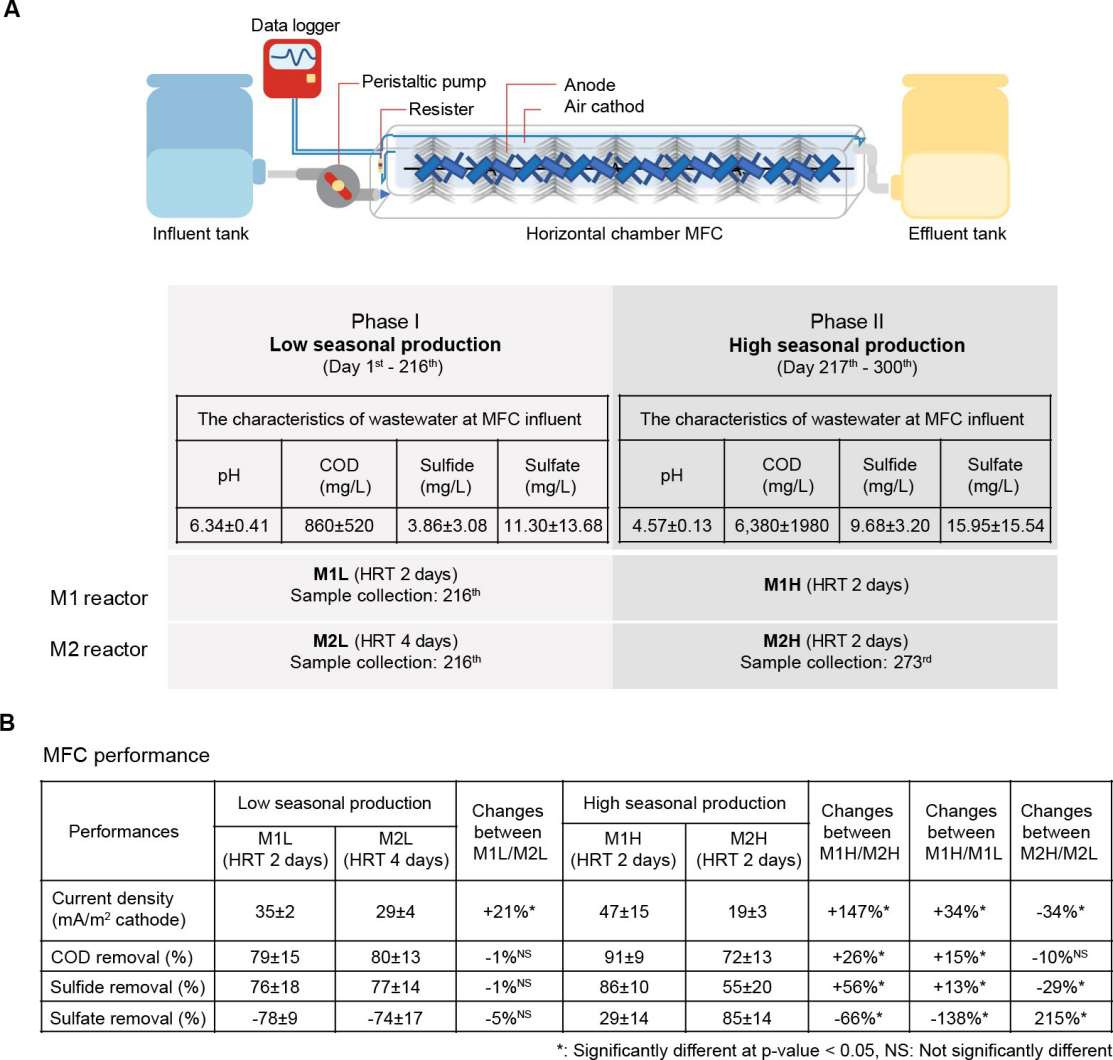
MFC system performance under low and high seasonal production at a canned-pineapple factory. **A**, A schematic diagram of a horizontal flow MFC bioreactor featuring key components such as an anode electrode, air-cathode, and external resistance is shown in the upper panel. The wastewater characteristics at low and high seasonal production and an overview of the MFC operation conditions are presented in the lower panel. **B,** The performance metrics of all MFC reactors (M1L, M2L, M1H, and M2H) as well as their comparative analysis.

In this study, two modules of horizontal single-chamber air-breathing MFCs were used (called M1 and M2). The MFC configuration is shown in Fig 1A (upper panel). Each MFC module consisted of two equal channels with a volume of 7.5 L each. The anode electrode was manufactured from carbon fiber and coated with palladium. The cathode electrode was made from carbon cloth and activated carbon, based on the United States patent US 8846220B2 [22]. The projected surface area of the cathodic electrodes was 0.038 m^2^. The proton exchange membrane was graphite-coated with a polymer (FLA-1005 PFSA dispersing 5% in water, EW, Fumatech, Bietigheim-Bissingen, Germany). The anode and cathode were connected to an external resistance of 200 Ω and a multichannel data logger (Graphtec Midi Logger GL820, Japan) for daily voltage measurements. Wastewater entered the system from a lower point located at the front and exited from an upper point at the far end toward the rear of the system. Sludge from lab-scale anaerobic digestion of molasses stillage was used as an inoculum in the anodic chamber at a concentration of 10 g VSS/L. The effluent obtained after treating canned pineapple processing (CPP) wastewater in a modified covered anaerobic lagoon (MCAL) served as the MFC influent, and its characteristics depended on the removal efficiency of MCAL and the production volume of CPP. The characteristics of the wastewater at low and high seasonal production levels are shown in Fig 1A (lower panel). During high seasonal production, the wastewater concentrations of COD, sulfide, and sulfate were significantly increased, whereas the pH dropped to *4*.*57±0*.*13*, necessitating its adjustment to a range of 5.0–6.0 using Na_2_CO_3_.

### MFC operating conditions

The MFC systems were setup at a canned-pineapple factory in Ratchaburi province, Thailand. The systems were operated at ambient temperatures ranging from 25 to 35°C and were continuously fed with the CPP wastewater for 300 days.

Because of fluctuations in the wastewater characteristics during low and high seasonal production levels, the experiments were separated into two phases. In the first phase (Phase I), the MFCs were operated continuously for 216 days to determine the optimum HRT for MFC operation at a low organic loading rate (OLR). Here, MFC reactors M1 and M2 (denoted as M1L and M2L) were operated at HRTs of 2 and 4 days, respectively. In the second phase (Phase II), the experiment continued from the 217^th^ day to the 300^th^ day during the high seasonal production level. The M1 and M2 reactors (denoted as M1H and M2H) were run at an HRT of 2 days to investigate their response to an elevated OLR. Details of the MFC’s operational settings are shown in Fig 1A.We conducted a comparative assessment of MFC performance, evaluating their efficiency in removing COD, sulfate, and sulfide, as well as their electricity generation (Current density) capacity. The MFC influent and effluent samples were collected weekly for the analysis of pH, COD, sulfide, and sulfate concentrations. The pH was measured using a pH meter (pH7110, WTW). COD was analyzed by a close reflux method using the HACH COD reagent, HACH DRB 200 and HACH DR 2700. Sulfide and sulfate were analyzed by the iodometric and turbidimetric methods, respectively, according to APHA standard methods (APHA, 2005). The electric parameters of the cells were determined as previously described by Lóránt et al. (2021) [23]. The cells’ internal resistance of the developed MFC systems was around 200 Ohm. The open circuit voltage of the systems was between 0.4–0.6 Volts. The voltage across the electrode was monitored over time using a digital multimeter (Yokogawa, Japan). The Wilcoxon non-parametric test was used to compare the performance of reactors, considering a significant difference at a *p*-value < 0.05.

### DNA extraction and 16S rRNA gene amplicon sequencing

A total of 14 samples (Fig 1A) were collected for the microbial community analysis. The sludge inoculum (from our laboratory collection) and effluent from the MCAL-treated CPP wastewater were used as the initial microbial data. On the 216^th^ day, eight samples were collected from reactors M1L and M2L to investigate microbial changes by the end of the low seasonal production. Additionally, four samples from reactor M2H were collected on the 273^rd^ day to investigate the microbial community during high seasonal production. For both reactors M1 and M2, three samples were obtained from anode-attached microorganisms at the inlet, middle, and outlet points. In addition, one sample of suspended microorganisms was collected.

Total DNA samples were extracted using DNeasy PowerSoil Pro Kit. The V3-V4 regions of the 16S rRNA genes were amplified. Next, the DNA libraries were generated with the NEBNext^®^ Ultra^TM^ DNA Library Prep Kit, and then sequencing was conducted using the Illumina HiSeq platform with 2 x 250 bp paired-end sequencing.

### 16S rRNA gene amplicon sequencing analysis

Amplicon sequencing samples were analyzed using the MiSeq SOP Mothur pipeline (v.1.40.5) [24]. Firstly, low-quality reads were filtered out, including (1) reads outside the target region of 384–431 bp (2) reads containing homopolymers > 8 bp, (3) reads containing ambiguous bases with quality scores lower than 25, and (4) reads containing chimeric sequences. Sequences that passed these quality filters were then clustered into OTUs with 97% similarity. Next, each OTU was taxonomically classified using the *k*-nearest neighbor consensus approach with the SILVA database (v.132) [25]. Subsequently, an OTU count table was generated for downstream analysis.

The diversity of the microbial community within OTUs was investigated. Four intra-sample diversity indices were applied to measure species richness and evenness, namely Chao1, ACE, Shannon-Weiner, and Simpson’s indices. The microbial composition was explored by PCoA with the Bray-Curtis dissimilarity index, using the phyloseq package [26]. High-abundance microbes (≥ 1% relative abundance) at both the phylum and genus levels were visualized and compared using ggplot and ggVennDiagram.

### Differential microbial abundance analysis

To compare microbial abundance between reactors, including the anodes and microbial suspension, OTUs with a read count of ≥ 4 reads in at least 3 samples were selected for further normalization using the TMM method. Testing for significant differences between conditions was conducted using Fisher’s exact test via edgeR [27] with an FDR of ≤ 0.05 and an absolute log_2_ fold-change of ≥ 5 for M2H/M2L and M1L/M2H, and ≥ 2 for M1L/M2L.

### Microbial association analysis

Putative microbes related to the desired MFC performance indices, namely (1) high current density generation and (2) high sulfide removal, were proposed based on association analysis. Firstly, we identified the predominant microbes, OTUs with significantly high abundance in M1L/M2L and M1L/M2H, both at the anode (A1, A2, A3) and reactor (A1, A2, A3, S) scales based on the set criteria, i.e., FDR < 0.05 and an absolute log_2_ fold-change ≥ 2 for M1L/M2L and ≥ 5 for M1L/M2H. If these microbes did not display strong correlations in terms of abundance profiles between M1L and M2L or M2H reactors, as determined by Spearman’s rank correlation with |ρ| < 0.8, they were considered to be predominantly associated with M1L, meaning they are more specific to the M1L reactor compared to the M2L or M2H reactors. Secondly, these microbes were further studied to explore the links between their average abundance in each reactor and MFC performance in terms of the (1) current density and (2) sulfide removal rate, via Spearman’s rank correlation.

## Results and discussion

### MFC performance at different HRTs

**Fig 2:**
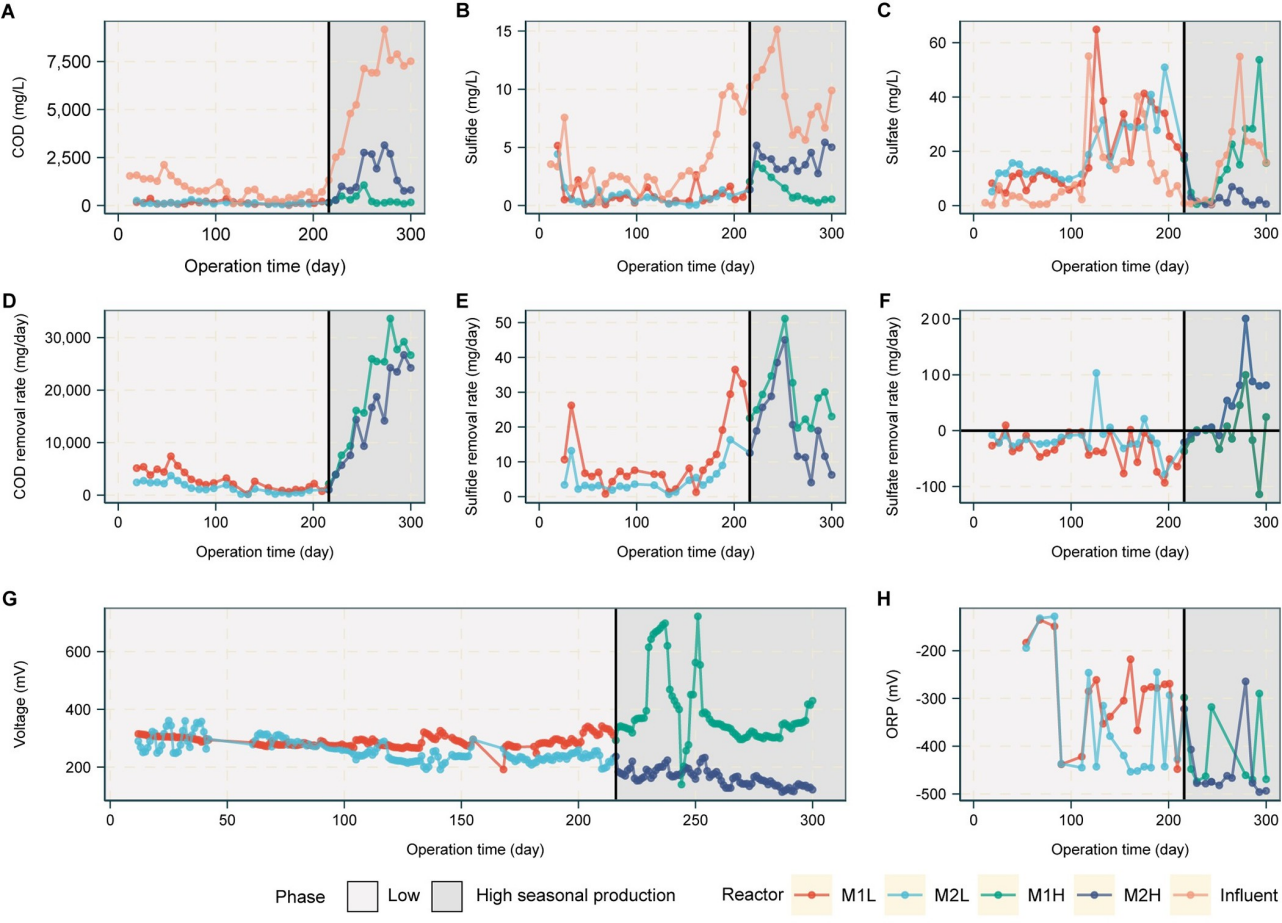
MFC performance evaluation over 300 days: **A,** COD concentration, **B**, sulfide concentration, **C**, sulfate concentration, **D**, COD removal rate, **E**, sulfide removal rate, **F**, sulfate removal rate, **G**, voltage, and **H**, oxidation-reduction potential (ORP) values.

**Fig 3:**
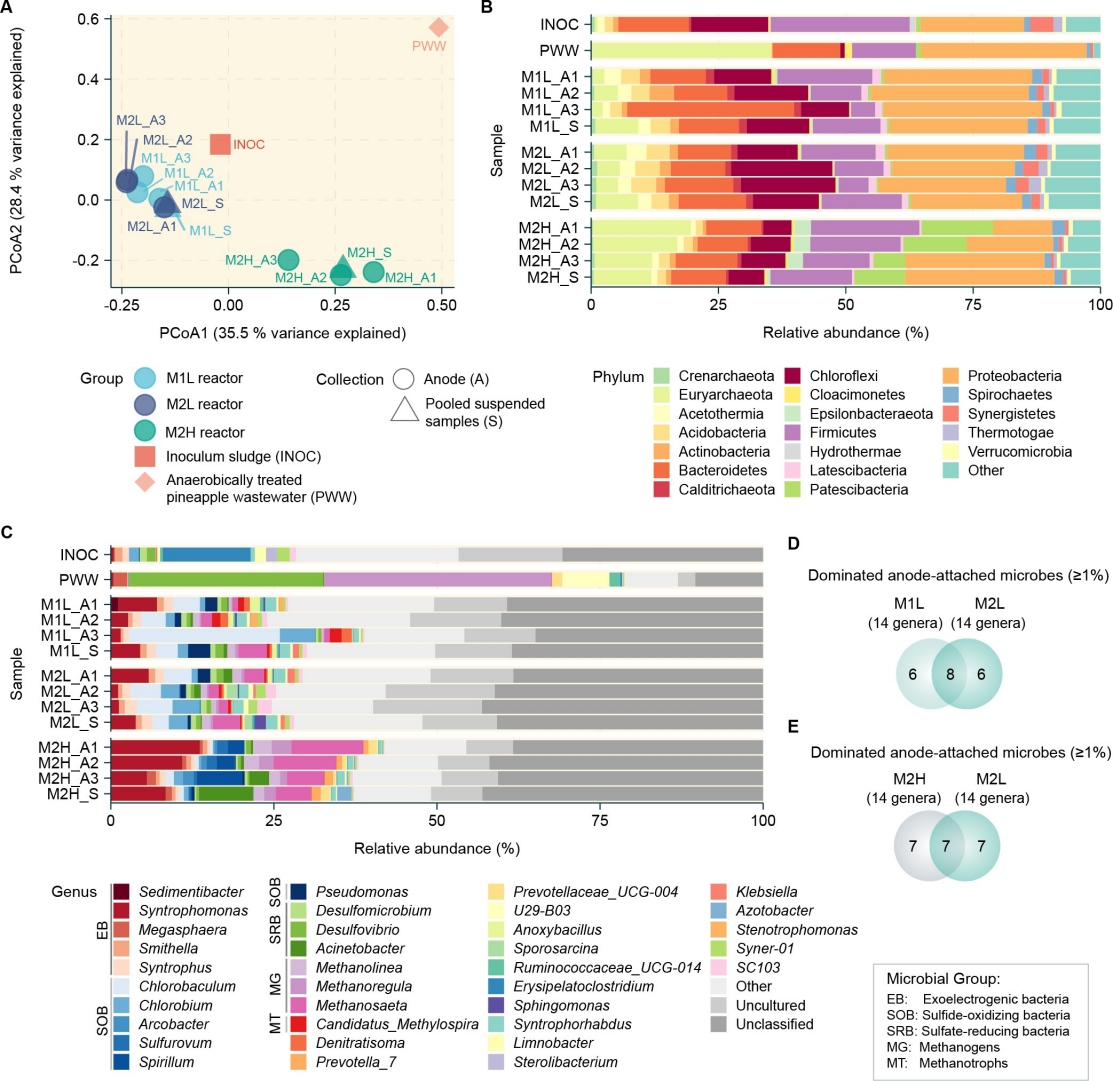
Characteristics of microbial communities in the MFC systems: **A**, Principal coordinate analysis (PCoA) using the Bray-Curtis dissimilarity. Microbial community profiles of MFC systems at the phylum (**B**) and genus (**C**) levels. Venn diagrams comparing anode-attached microbial genera in M1L and M2L (**D**) and M2L and M2H reactors (**E**). All microbiome samples in MFC systems were collected from anodes attached at the inlet (A1), middle (A2), and outlet (A3) points of the reactor, pooled suspended samples (S) of M1L, M2L, and M2H reactors, as well as the inoculum sludge (INOC) and anaerobically-treated pineapple wastewater (PWW).

The hydraulic retention time (HRT) is a primary operating parameter that plays a crucial role in optimizing the performance of MFCs, particularly when confronted with variable wastewater characteristics. Adjusting the HRT involves a systematic readjustment process aimed at controlling the organic loading within the MFC environment. This strategic adaptation allows for more effective management of MFC performance in response to changing wastewater conditions. The MFC reactors, which were fed with the AD effluent, operated at different HRTs of 2 days (M1L) and 4 days (M2L). These variations in HRTs were applied to treat industrial wastewater during the low-seasonal production phase (Phase I), resulting in M1L experiencing twice the OLR compared to M2L. Fig 1B presents the performance of the MFCs in terms of the removal efficiency and current density. The analysis revealed that both MFCs exhibited nearly identical COD and sulfide removal efficiencies. However, a notable difference was observed in their electricity generation (i.e., current density). M1L surpassed M2L in electricity production owing to its superior COD and sulfide removal rates. Remarkably, M1L achieved these results while operating at double the organic and sulfide loading compared to M2L (Fig 2D and 2E). This outcome is consistent with the overall microbial profile shown in the PCoA graph in Fig 3A, which demonstrated a similarity between M1L and M2L. It is, therefore, possible that the microorganisms in both reactors retained their metabolic capacity to remove both the organic and inorganic substrates even when the organic loading rate was doubled from 0.22 to 0.43 gCOD L^-1^ d^-1^. M1L and M2L could remove about 80 percent of COD and up to 77 percent of sulfide. These MFCs showed greater COD removal efficiency compared to a double-chamber MFC utilizing synthetic wastewater [28] and a pilot‐scale stacking tubular MFC system designed for swine wastewater [29].

The variations in operating conditions between M1L and M2L did not significantly impact the quality and composition of the effluents they generated, as indicated by similarities in their concentrations of COD, sulfide, and sulfate: 148±78 mg/L, 0.99±1.06 mg/L, and 20.07±14.86 mg/L for M1L, and 136±61 mg/L, 0.82±0.88 mg/L, and 19.67±11.30 mg/L for M2L, respectively (Fig 2A–2C). Additionally, the pH and oxidation-reduction potential (ORP) under these operating conditions did not vary significantly. Specifically, the observed pH values of M1L and M2L effluents were 6.96±0.12 and 6.92±0.18, respectively, while their corresponding ORP values were -293±88 mV and -351±114 mV (Fig 2H).

The voltage obtained from M1L and M2L was in the range of 192–342 mV (Fig 2G). This is comparable to findings in previous studies with real wastewater, organic- rich chocolate factory wastewater and domestic wastewater [11, 30]. The current density in M1L was 21% higher than in M2L (Fig 1B). This higher current density obtained in M1L was likely caused by the twofold increase in COD and sulfide conversion rates (Fig 2D and 2E). The COD and sulfide removal occurred through the oxidation of organic compounds and sulfide, resulting in the generation of electrons and protons as products. The two-times higher flow rate in M1L led to more efficient transportation of generated electrons and protons within the system, contributing to the higher current density observed in M1L compared to M2L.

Furthermore, electricity generation might also be related to the increased sulfate concentration in the effluents of both M1L and M2L (Fig 2C). Sun and his team reported that the oxidation of sulfide to $S^{0}/S_{x}^{2-}$ and further to $S_{4}O_{6}^{2-}/S_{2}O_{3}^{2-}$ occurred spontaneously as electrochemical reactions, leading to electricity generation [31]. The formation of $S^{0}/S_{x}^{2-}$ and $S_{2}O_{3}^{2-}$ was accelerated by the bacteria in the MFC anode, while ${SO}_{4}^{2-}$ was generated through microbial catalysis. The microbe-assisted production of $S_{2}O_{3}^{2-}$ and ${SO}_{4}^{2-}$ resulted in a sustained current in the MFC [31]. Therefore, the higher sulfate generation in M1L demonstrated that sulfide oxidation could occur better in M1L than in M2L (Fig 2E and 2F).

### MFC performance alteration due to increased OLRs during high seasonal production

In Phase II of the experiment (day 217–300), Both M1 and M2 faced increasing concentrations of COD, sulfide, sulfate due to high seasonal production (Fig 1A). In this phase, M1 and M2 were named M1H and M2H, respectively. Both MFCs were operated at the same HRT and OLR: M1H maintained a 2-day HRT, while that of M2H was reduced from 4 days to 2 days. In the case of M1H, the COD, sulfide, and sulfate loading rates were increased by 7.4, 2.4, and 1.4 times, respectively, compared to M1L Meanwhile, M2H exhibited even more substantial increases, with the COD, sulfide, and sulfate loading rates increasing by 12.8, 4.4 and 2.8 times compared to M2L. The performance of M2H might be affected by much greater changes in COD and sulfide loadings that would disturb the microbial community in the system. As shown in Fig 1B, M1H exhibited significantly higher COD and sulfide removal efficiency compared to M2H. Also, the current density of M1H surpassed that of M2H by 147%. The effluent quality, as measured by COD and sulfide concentrations, was notably superior in M1H compared to M2H. Specifically, the effluent from M1H showed COD, and sulfide concentrations of 300±264 mg/L, 1.37±1.16 mg/L, respectively, while that of M2H exhibited values of 1,480±1,028 mg/L, and 3.77±1.13 mg/L for COD, and sulfide concentrations, respectively (Fig 2A and 2B). Both systems had nearly identical observed pH values of effluent, with M1H at 7.01±0.20 and M2H at 6.98±0.20. M1H exhibited a significantly higher voltage compared to M2H, with values of 385±120 and 164±29 mV, respectively (Fig 2G).

Increasing the OLR stimulated the COD removal rate of both MFCs (Fig 2D) and generated a high amount of electrons, as can be seen from the highly negative ORP values (Fig 2H). However, sulfide oxidation occurred more efficiently in M1H than in M2H. These results suggested that sulfide oxidation played a significant role in M1H, contributing substantially to electricity generation. As previously discussed, the degree of sulfate generation resulting from sulfide oxidation had a direct impact on electricity generation. Hence, the higher sulfate concentration observed in the effluent of M1H serves as an indicator of the enhanced efficiency of sulfide oxidation in this system compared to sulfate reduction. In contrast, sulfate reduction progressed more efficiently in M2H than in M1H. It should be noted that heightened sulfate reduction can adversely affect electricity generation, as sulfate-reducing bacteria (SRB) consume both the organic matter and electrons generated during the process, which can explain the reduced electron transport to the anode electrode. In addition, the lower current density observed in M2H might be because of inefficient sulfide oxidation, as can be seen from the higher sulfide concentration in Fig 2B. These indicate the different mechanisms between the two MFCs. Thus, M1, which previously operated at an HRT of 2 days, could more efficiently adapt to changes in organic loading compared to M2. Different HRTs led to variations in the composition and structure of the microbial communities within the MFCs, significantly impacting their performance.

The responses of M1 to increases in COD and sulfide concentrations were investigated. We found that as the OLR increased, there was a corresponding improvement in both the electricity generation and chemical removal efficiency of the system. The current density, COD removal efficiency, and sulfide removal efficiency of M1H significantly increased by 34%, 15%, and 13%, respectively, relative to M1L. In general, the OLR profoundly impacted the MFC performance by affecting substrate degradation in the anodic chamber and electron transportation to the anode [11]. As shown in Fig 2, both M1L and M1H relied on organic and sulfide oxidation as their primary mechanisms. In addition, as the OLR increased, higher sulfate reduction was observed, as indicated by the higher sulfate removal efficiency observed in M1H. The higher sulfate concentration in the effluents of both M1L and M1H could be attributed to sulfate originating from sulfide oxidation as well as from the influent. However, M1H exhibited a lower ratio of sulfate produced to sulfide removed (0.14) compared to M1L (2.83), as shown in Fig 2C and 2F. This lower ratio suggests that sulfide was oxidized to elemental sulfur or thiosulfate instead of sulfate, contributing to the high electricity generation in the MFC system [32].

Meanwhile, the M2 reactor was perturbed by decreasing the HRT and increasing the OLR. When comparing M2L and M2H, with the latter having an OLR 12.8 times higher, the current density of M2H was 34% lower than that of M2L. Furthermore, M2H exhibited a 29% lower sulfide removal efficiency, but a remarkable 215% higher sulfate removal efficiency. Thus, increasing OLR while decreasing HRT strongly affected the performance of M2. The results also suggested a shift in the main mechanism of M2 from sulfide oxidation to sulfate reduction when OLR was increased. In addition, since the sulfide removed by sulfide oxidation could be regenerated by sulfate reduction, lower sulfide removal was obtained in M2H. Besides, increased sulfate reduction in the system resulted in lower electricity generation as the electrons generated within the system were consumed by SRB. This result indicates that sulfate outcompeted the anode as an electron acceptor, resulting in lower electron transport to the anode electrode.

In this study, the promising efficiency of the M1 system in improving water quality and harnessing surplus energy was highlighted. Applying MFCs instead of using the existing post-treatment systems like stationary ponds and aerated lagoons could reduce operational costs, generate electricity, and prevent the release of sulfide, which is harmful to living organisms, including humans, and causes odor problems. Therefore, this biorational approach that incorporates MFCs with an AD system represents a sustainable wastewater treatment solution for the canned pineapple processing industry. This approach can promote positive net energy generation and holds promise for practical implementation on an industrial scale.

### Analysis of microbial community in the operating MFC

Microbial communities in the MFCs were investigated to understand how their functions are linked to the performance of the system following changes in the operational HRT and OLR. Microbial comparisons within and between the MFC reactors were performed using a metagenomic approach. The amplicon sequencing reads from each sample were preprocessed and clustered into OTUs (S1 and S2 Tables). The microbial diversity was examined to understand how the ecological properties of the microbiome influence the MFC performance.

The microbial community diversity was investigated at the OTU level within different parts of the system, namely the MFC inoculum, the anaerobically-treated pineapple wastewater (PWW), and both the anodic and suspension components of the MFC reactors, under low and high OLRs. Alpha-diversity indices such as Chao1, ACE, Shannon, and Simpson’s indices were used to estimate the richness and/or evenness of the microbial communities. Notably, the microbial communities within the MFC reactors and inoculum were more diverse and richer than those in the PWW (S3 Table). Besides, MFC reactors operating at low OLR (M1L and M2L reactors) showed slightly higher richness and evenness compared to the starter inoculum. Interestingly, the microbial community within M2H, which operated under high organic loading, showed lower richness and evenness than that of MFCs operated at low organic loading.

The similarity of the microbiome across the samples was determined based on the Bray-Curtis index, and the visualization was done via PCoA (Fig 3A). The microbial communities within the MFC reactors, specifically the anode and suspension, were analyzed under different operational conditions. The microbial communities in M1L and M2L were more closely related to the inoculum than to the influent wastewater (PWW), suggesting that the microbiome in the inoculum played an important role as active biocatalysts for anaerobic digestion in the MFC systems (Fig 3A). Thus, inoculum selection is critical to the MFC operational success. Although M1L and M2L were operated at low seasonal production and different HRTs, their microbial composition was relatively similar. In contrast, the microbial composition of M2H, which was operated under high seasonal production with an OLR over seven times higher than that of M1L, showed greater dissimilarity to M1L notwithstanding their similar HRTs (Fig 3A). Therefore, OLR had a greater effect on the microbial community with the MFCs than HRT. The analysis revealed the presence of eight predominant microbial genera in the inoculum, each with a relative abundance greater than 1%. The genera were *Erysipelatoclostridium* (13.48%), *Syner-01* (1.91%), *Sterolibacterium* (1.72%), *Limnobacter* (1.72%), *Chlorobium* (1.51%), *Desulfovibrio* (1.27%), *Smithella* (1.16%), and *Desulfomicrobium* (1.12%). Six predominant microbial genera, each with a relative abundance greater than 1%, were found in PWW, namely *Methanoregula* (34.81%), *Desulfovibrio* (29.68%), *U29-B03* (7.18%), *Megasphaera* (2.16%), *Ruminococcaceae_UCG-014* (1.67%), and *Prevotellaceae_UCG-004* (1.57%). Among them, *Desulfovibrio* was predominant in both the inoculum and influent PWW (Fig 3C).

### Microbial community dynamics at varying HRTs

MFCs operating at both low (M1L) and high (M2L) HRTs showed similar levels of organic removal efficiency (COD, sulfide, and sulfate removal efficiency) during low OLR operation (0.22 and 0.43 gCOD L^-1^ d^-1^ for M2L and M1L, respectively). However, both differed in their ability to produce electricity (current density). Analysis of microbial communities under different HRTs revealed key microbial groups that may be relevant to the performance of the M1L and M2L reactors, based on their predominance. A total of 20 genera with a relative abundance of ≥1% dominated the anode-attached microbes in either M1L or M2L in at least one sampling position (inlet, middle and outlet) (Fig 3D). Furthermore, eight common genera dominated the anode-attached samples of both M1L and M2L, namely *Syntrophomonas*, *Syntrophus*, *Chlorobaculum*, *Chlorobium*, *Pseudomonas*, *Methanolinea*, *Methanosaeta*, and *Syntrophorhabdus*. Six genera were specific to anode-attached samples of M1L, namely *Smithella*, *Denitratisoma*, *Candidatus Methylospira*, *Anoxybacillus*, *Sedimentibacter*, and *Stenotrophomonas*, and six were specific to anode-attached samples of M2L, namely *Desulfovibrio*, *Acinetobacter*, *Klebsiella*, *SC103*, *Sporosarcina*, and *Syner-01* (Fig 3C and 3D).

**Fig 4:**
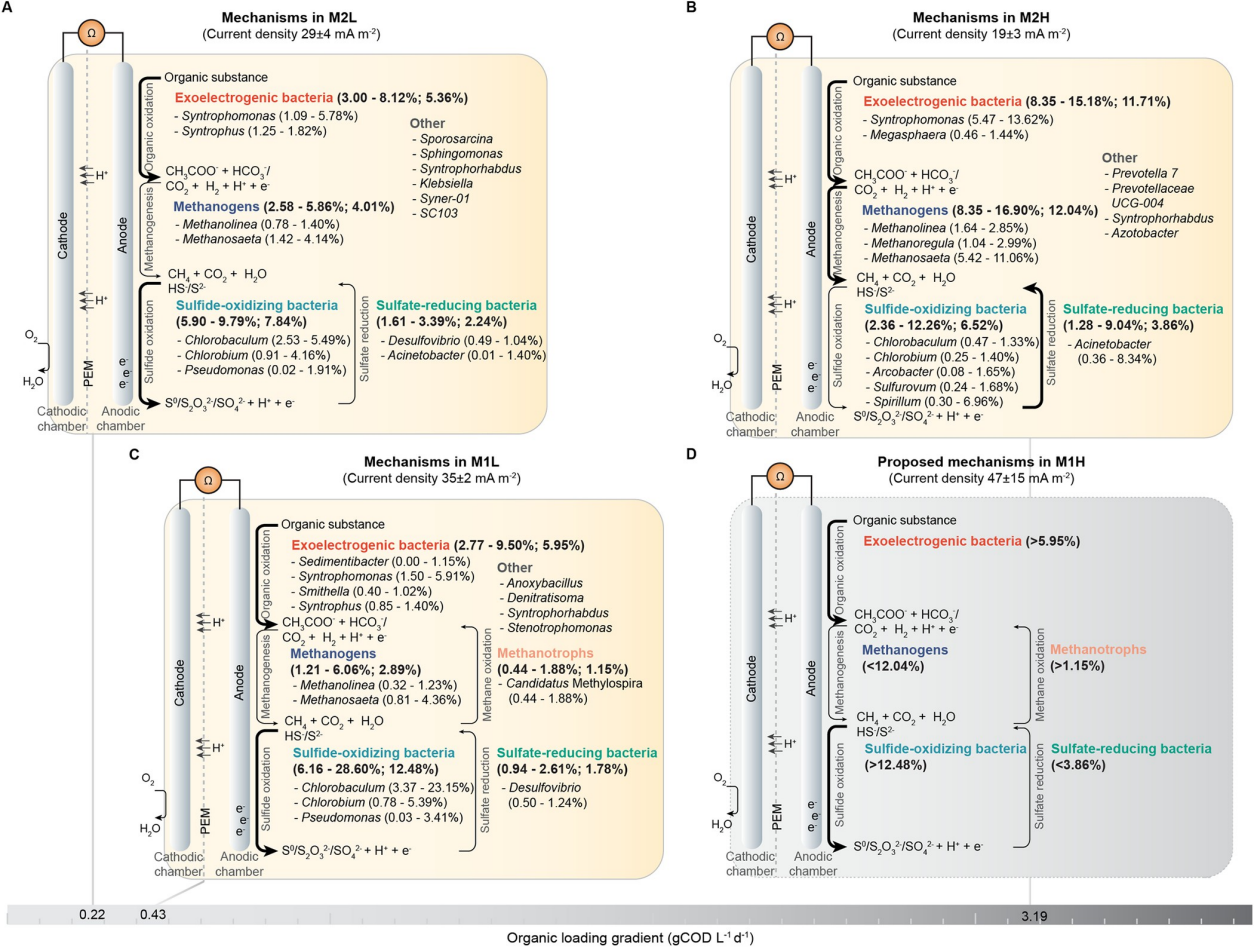
Proposed metabolic activities based on microbial abundance profiles of each MFC reactor. M1H has the highest current density (**D**) followed by M1L (**C**), M2L (**A**), and M2H (**B**). The range and average microbial abundance are reported in parenthesis for M1L, M2L, and M2H, while estimates for M1H are based on its performance. The predominant genera in each group are indicated along with their abundance.

The microbes were grouped into five main functional groups based on their metabolic activities, namely electrogenic bacteria (EB), sulfide-oxidizing bacteria (SOB), sulfate-reducing bacteria (SRB), methanogens (MG), and methanotrophs (MT) (Fig 4 and S4 Table). A predominance of EB was observed in both M1L (2.77–9.50%) and M2L (3.00–8.12%), with members including *Syntrophomonas*, *Syntrophus*, *Sedimentibacter* and *Smithella* (Fig 4A and 4C). Electrogenic bacteria can oxidize complex organic compounds in wastewater, converting them to volatile fatty acids (VFAs) or smaller molecules [33]. *Syntrophomonas*, *Syntrophus*, and *Smithella* are cross-feeding, energy-producing and, possibly, electron-donating bacteria that grow in partnership with other microbes [34, 35]. The distinct dominant genera of EB might be related to the higher voltage in M1L compared to M2L. Interestingly, the relative abundance of SOB in M1L (6.16–28.60%) exceeded that in M2L (5.90–9.79%), which corresponded to the two times higher sulfide removal rate in M1L (Fig 2D). The twice higher organic loading rate in M1L relative to M2L likely benefited the growth of SOB by increasing the organic and inorganic substrates, especially carbon and sulfide sources. Feng et al. (2010) showed that *Chlorobaculum tepidum*, a photosynthetic green sulfur bacterium, could grow on mixotrophic cultures containing acetate or pyruvate [36]. Furthermore, our microbiome analysis results also demonstrated the enrichment of SOB in M1L, especially the dominant *Chlorobaculum genus* with a relative abundance range of 3.37–23.15% in M1L compared to its range of 2.53–5.49% in M2L (Fig 4A and 4C). This SOB enrichment in M1L may be a consequence of the increased availability of organic and inorganic substrates in the wastewater. Therefore, the higher electricity generation in M1L is possibly linked to the higher recovery of electrons from sulfide [37].

Besides the genus-level analysis of the microbial abundance, OTU differential abundance among anode-attached microbes in M1L and M2L was further investigated (S1A–S1C Fig). A total of 46 significant differentially abundant OTUs were identified. Interestingly, 42 OTUs among them were highly abundant in M1L, whereas only 4 OTUs were highly abundant in M2L. This could be because of the higher organic loading in M1L. M1L had a higher abundance of EB like *Sedimentibacter* (OTU00905 and OTU00253), which can grow on amino acids and produce butyrate, propionate, acetate, and sulfide [38]. Furthermore, the higher electricity generation in M1L might be related to the elevated abundance of *Denitratisoma* (OTU00118 and OTU00080), which is a nitrate-reducing genus [39]. The oxidation of sulfide to ${SO}_{4}^{2-}$ or elemental sulfur (S^0^) when coupled with nitrate (NO^3−^) reduction can potentially produce fourteen electrons ($12HS^{-}+2{NO}_{3}^{-}\to 12S^{0}+N_{2}+6H_{2}O+14e^{-}$) [40]. However, to accurately confirm this phenomenon, analysis of nitrate levels and their changes during the MFC operation was taken into account. The significantly higher abundance of MT (0.44–1.88%), including aerobic methane-oxidizing bacteria such as *Candidatus Methylospira* (OTU00717) and *Rhizobiales* (OTU00312), in M1L suggests that MT might be a key player in electricity generation in MFC systems. MT can utilize methane and plays a pivotal role in extracellular electron transfer via outer membrane *c*-type cytochromes [41]. Recently, the complete genome sequence of *Methylospira mobilis* was annotated, revealing protein-encoding genes functioning in the complete electron transport chain and two ATP synthase operons [42]. This suggests the involvement of MT in electron transfer within MFC systems. Furthermore, a significantly higher abundance of *Clostridium* (OTU00607, OTU00515, and OTU629) was found in M1L relative to M2L. *C*. *acetoburylicum* can oxidize acetate to produce electrons [18]. Thus, the higher abundance of EB, SOB, and MT in the M1L system may have contributed to the higher electric generation observed in M1L, compared to M2L.

### Microbial community changes in response to higher OLR during high seasonal production

As described earlier, changes in operational conditions, characterized by a lower HRT and a higher OLR, had a negative impact on the electricity generation efficiency of M2H, leading to a decrease in its performance compared to M2L. Moreover, the changes in operational conditions led to a shift in the primary microbial mechanism of M2, changing from sulfide oxidation in M2L to sulfate reduction in M2H, accompanied by corresponding changes in the microbial communities. As shown in Fig 4A, SOB were the dominant microbial group in M2L, accounting for 5.90–9.79% of the microbial population. This group consisted of the genera such as *Chlorobaculum*, *Chlorobium*, and *Pseudomona*s, which are directly involved in sulfide oxidation and electricity generation in MFCs [32]. The increase in organic loading in M2H led to a substantial rise in the abundance of MG and SRB (Fig 4B). Methanogenic archaea were the predominant microbial group in M2H, representing 8.35–16.90% of the community, and included the genera *Methanolinea*, *Methanoregula*, and *Methanosaeta*. Moreover, the abundance of SRB in M2H exceeded that in M2L, with levels ranging from 1.61–3.39% in M2L compared to 1.28–9.04% in M2H. The increase in populations of MG and SRB found in M2H might be due to the elevated concentration of their primary food source, short-chain volatile fatty acids (VFA), in the wastewater. This increase occurred during periods of high seasonal production at the factory, leading to VFA levels equivalent to 4,980 mg COD/L. It has been reported that acetate and sulfate favor the growth of *Methanosaeta concilii* and *Desulfobacter latus*, which are co-existing aceticlastic microbes found among MG and SRB [43]. MG and SRB cannot generate free electrons as they are not EB; however, they consume electrons in the AD system. MG consume substrates such as acetate and H_2_, generating methane as a byproduct. Meanwhile, SRB utilize sulfate as an electron acceptor, producing elemental sulfur or sulfide. The findings indicate that high organic loading significantly enhanced the growth of MG and SRB, leading to their dominance in electron consumption over the anode.

Differential OTU abundance of anode-attached microbes in M2L and M2H was analyzed to explore microbiome changes linked to MFC performance. As demonstrated in S2A–S2C Fig, not only was a change in the microbial community observed, but their abundance also affected the electricity generation of MFC. The higher abundance of the anaerobic SOB *Paracoccus* (OTU00211) in M2L may be related to the high current density observed in the system. *Paracoccus* can thrive on one-carbon compounds such as CO_2_ or formate and is involved in thiosulfate oxidation, generating free-electrons as a final product [44]. On the other hand, the significantly higher abundance of *Sulfurovum*, another SOB genus, in M2H may have contributed to the observed low current density in the system. *Sulfurovum* primarily grows chemolithoautotrophically using hydrogen, elemental sulfur, and thiosulfate as electron donors and oxygen, nitrate, thiosulfate, and elemental sulfur as electron acceptors, with CO_2_ as the carbon source [45]. In addition, the higher abundance of MG and SRB in M2H indicated that organic removal in M2H was mainly associated with methanogenesis and sulfate reduction. An increase in OLR with a high COD-to-sulfate ratio promoted the growth of MG and SRB, resulting in low electricity generation [46]. The increase in MG such as *Methanosarcina* (OTU00302) and *Methanobacterium* (OTU00346), which can utilize acetate and *H*_2_ as substrates, enhanced the utilization of organic substances in PWW for methane production. Also, the significant increase in SRB such as *Acinetobacter* (OTU00030) and *Sulfurospirillum* (OTU00632) in M2H strongly correlated with the high COD and sulfate removal efficiencies observed in M2H (Fig 1B and S2A–S2C Fig). SRB can utilize organic matter as an electron donor and sulfate as an electron acceptor, generating sulfide as a product via cellular respiration [47]. The significantly higher abundance of EB such as *Syntrophomonas* (OTU00124 and OTU00389) and *Syntrophomonadaceae* (OTU00684 and OTU00685) in M2H indicates the well-established interspecies electron transfer between syntrophs and MG. These EB are known for their beta-oxidization of saturated fatty acids to acetate or acetate and propionate [48], contributing to the overall microbial interactions and electron transfer processes within the MFC system.

M1H showed the highest performance among all the measured parameters, including current density, COD removal, and sulfide removal (Fig 1B). Its OLR increased from 0.43 to 3.19 gCOD L^-1^ d^-1^ during high seasonal production (Fig 4C and 4D). Comparing the performance of M1H and M1L, M1H had higher current density and superior COD and sulfide removal rates, with improvements of 34%, 15%, and 13%, respectively. Between M1L and M2L, the former showed 21% higher current density and a higher abundance of SOB, MT, and EB (Fig 4A and 4C). The difference in microbial abundance between M1H and M1L, characterized by a higher presence of SOB, EB and MT in M1H, and a lower abundance of MG and SRB than in M2H is shown in Fig 4D. At the initial stage, the OLR proved to be an important parameter for optimizing MFC performance. M1H could better tolerate perturbation from the substantially high loading rate during high seasonal production while maintaining its performance, and it showed improved performance as the OLR increased significantly. By contrast, when M2H was operated at the same OLR and HRT as M1H in the second phase, it suffered a significant decrease in performance across all parameters: current density, COD removal, and sulfide removal, when compared with M2L. Actually, M2H showed the lowest performance among others.

### The role of microbiota in electricity generation and sulfide removal

To determine key microbes specific to M1L (the best operating condition for microbial sample analysis) and establish their link to MFC performance, including electricity generation and sulfide removal, we conducted an association study between microbial abundance and MFC performance. Firstly, the predominant microbes specific to M1L were identified by comparing microbial abundance under two different perturbation conditions, both at the anode and reactor scales: (1) a comparison between HRTs of 2 days and 4 days (M1L and M2L) and (2) a comparison between low OLR (M1L) and high OLR (M2H) setups. Two criteria were employed for the comparison, namely 1) significantly higher abundance in M1L and 2) inconsistency in abundance when compared to the M2L and M2H conditions.

**Fig 5:**
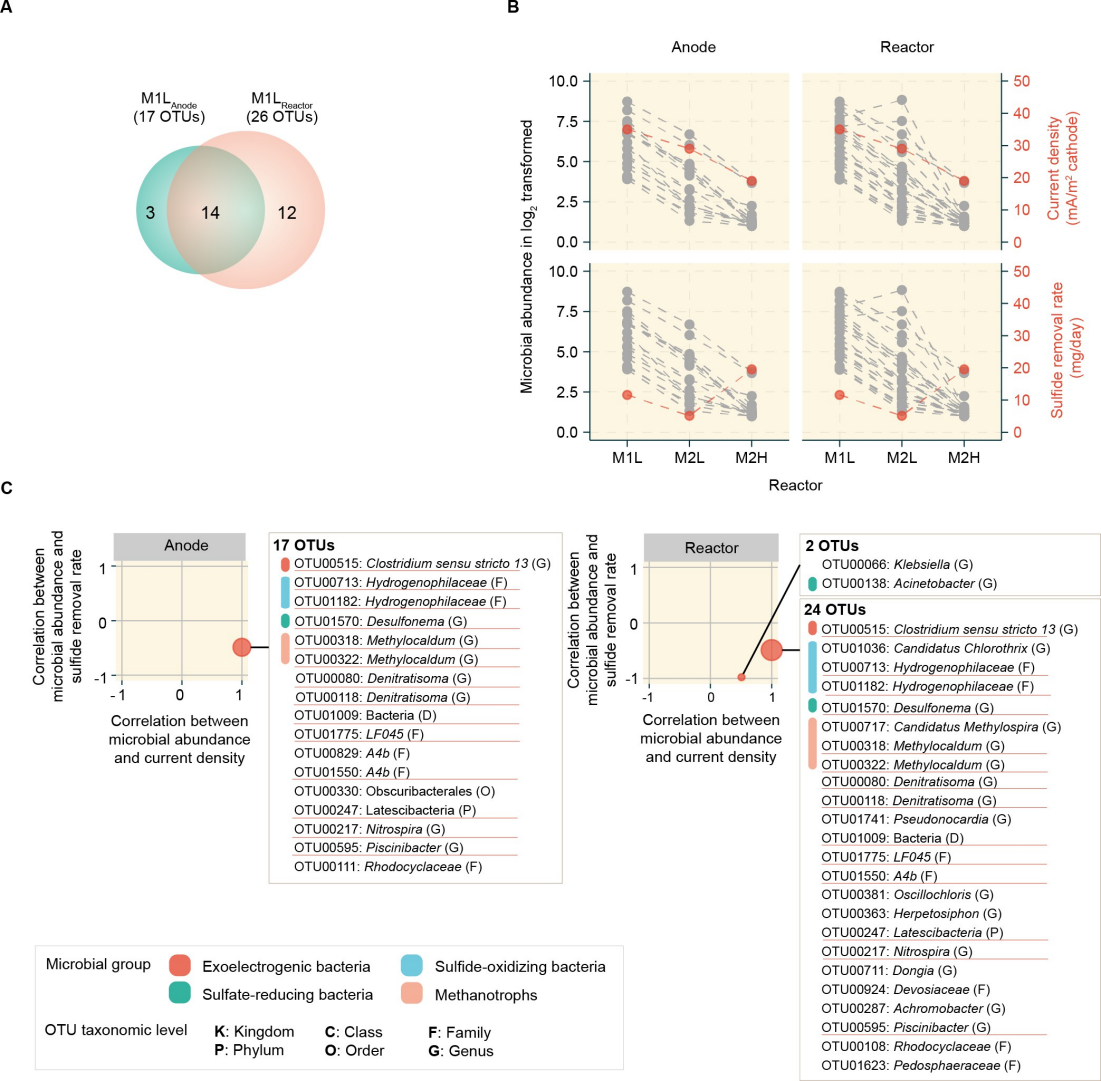
M1L-specific microbes potentially related to MFC performance. **A**, Venn diagram illustrating the distribution of all 29 microbes specific to M1L within both the anode-attached biofilms and reactor. **B**, The average microbial abundance, current density and sulfide removal rate for anodes and reactors in different systems. **C**, Association of microbial abundance with current density and sulfide removal at anode (left), and reactor (right) levels.

We identified 42 OTUs that were significantly higher in abundance in M1L than in M2L at the anode (A1, A2, A3) scale and 38 OTUs that were more abundant in M1L compared to M2L at the reactor (A1, A2, A3, S) level (S1 Fig). Additionally, 43 and 30 OTUs exhibited significantly higher abundance in M1L than in M2H at the anode (A1, A2, A3) and reactor (A1, A2, A3, S) scales (S3 Fig). These OTUs were further evaluated to identify inconsistencies in their abundance under M2L and M2H conditions, using Spearman’s rank correlation (S5 Table). Finally, a total of 29 predominant microbes specific to M1L were identified (Fig 5A). Among these, 17 OTUs were found at the anode scale, while 26 OTUs were found at the reactor scale. The average microbial abundance at both the anode (A1, A2, A3) and reactor (A1, A2, A3, S) scales for each MFC reactor was plotted against the two key MFC performance indicators, as shown in Fig 5B.

All 17 M1L-specific OTUs at the anode exhibited a Spearman’s rank correlation coefficient of one for the current density. Among them were one EB, *Clostridium* (OTU00515), two SOB, *Hydrogenophilaceae* (OTU00713 and OTU01182), one SRB, *Desulfonema* (OTU01570), and two MT, *Methylocaldum* (OTU00318 and OTU00322), as shown in Fig 5C. Nevertheless, these microbes did not exhibit a strong correlation with sulfide removal rate, as the transition from sulfide oxidation in M1L and M2L to sulfate reduction in M2H resulted in a sulfide-sulfate conversion.

For the reactor, a total of 26 OTUs were found. Among these, 24 OTUs showed a Spearman’s rank correlation coefficient of one for current density. These comprised all six functionally characterized OTUs at the anode, in addition to one SOB, *Candidatus Chlorothrix* (OTU01036), and one MT, *Candidatus Methylospira* (OTU00717) species. Therefore, increasing the abundance of these microbial species would increase electricity generation in the MFCs. One OTU, an SRB named *Acinetobacter* (OTU00138), was identified as having a negative correlation with the sulfide removal rate (Fig 5C (right)). An increase in the abundance of *Acinetobacter* (OTU00138) would decrease the sulfide removal rate in the MFC.

Analyzing the performance of the MFC system using real wastewater can be challenging due to the interplay of many factors that influence treatment efficiency and power generation. This work identified key microbes associated with current density and sulfide removal. Dynamic variations in EB, SOB, MT, and SRB affected the current density. *Klebsiella and Acinetobacter* were negatively correlated with the sulfide removal rate.

### Practical guidelines for maximizing MFC performance in industrial environments

In this work, MFCs were applied for the post-treatment of sulfide-rich wastewater from the canned pineapple industry, after anaerobic digestion (AD) treatment. The microbial community within the MFC reactor plays a key role in determining the overall operating performance. These microbes work in synergy to remove organic and inorganic contaminants in the influent, simultaneously producing electricity. Selection of the proper inoculum rich in MFC-supporting microbes is a key step in setting up an effective MFC operation. As revealed by principal coordinate analysis (Fig 3A), the microbial community profiles in the MFCs were more similar to those of the inoculum than the anaerobically treated pineapple wastewater. Hence, the desired functions of the microbial community in the MFCs could be designed by carrying out inoculum selection with diligence. Second, it is imperative to determine the optimal OLR for the MFC reactor, as this enables the formation of a robust MFC microbial community, enhancing the system’s tolerance to variable feed conditions resulting from seasonal variations in industrial production. As revealed in this study, the MFC with an HRT of 2 days (M1L) performed better than that with an HRT of 4 days (M2L) especially in electricity generation, with a 21% increase. Moreover, under a higher OLR, corresponding to high seasonal production, M1H performed better than the other MFC reactors in terms of electricity generation, COD removal, and sulfide removal (Figs 1B and 4). Third, it is essential to maintain the population of MFC-supporting microbes to ensure the stability of the overall microbial function and MFC performance throughout the operation period. Our analysis of microbial abundance under low seasonal production revealed that a higher abundance of EB, SOB and MT contributed to the superior performance of M1L, especially in electricity generation (Fig 4). To enhance MFC performance, it is recommended to maintain a high composition of EB such as *Clostridium* (OTU00515), SOB such as *Hydrogenophilaceae* (OTU00713 and OTU01182) and *Candidatus Chlorothrix* (OTU01036)), and MT such as *Methylocaldum* (OTU00318 and OTU00322) and *Candidatus Methylospira* (OTU00717)), while suppressing the activity of SRB such as *Acinetobacter* (OTU00138), as indicated by the association analysis (Fig 5C). These species represent valuable targets for microbial adjustment to maintain high MFC performance in the treatment of real industrial sulfide-rich wastewater.

## Conclusions

This study demonstrates the effectiveness of MFCs as a post-treatment system for removing organic and sulfide residues in AD effluents while also producing electricity. Here, the effects of HRT and OLR on the performance and microbiome dynamics of a horizontal single-chamber air-breathing MFC for treating wastewater generated during the processing of canned pineapple were evaluated. During the low seasonal production period, which was characterized by a low COD in the wastewater, the MFCs were operated at different HRTs (2 days for M1L and 4 days for M2L). M1L, despite operating at twice the OLR of M2L, showed superior performance, especially in electricity generation. When exposed to higher wastewater COD levels, during high seasonal production, and with a similar HRT of 2 days (OLR of 3.19 gCOD L^-1^ d^-1^), M1H outperformed M2H. Moreover, M1H achieved 47±15 mA/m^2^ current density, 91±9% COD removal efficiency, and 86±10% sulfide removal efficiency at the shortest HRT (2-day) and the highest OLR (3.19 gCOD L^-1^ d^-1^) among all four MFC reactors. For practicality purposes, the following guidelines are suggested for effective MFC application in industrial wastewater treatment: (i) the microbial inoculum is important in setting up the microbial community within the MFC, ultimately impacting the reactor performance, (ii) initiating the MFC operation with the proper OLR helps in forming a robust and effective microbial community capable of adapting to varying OLR conditions across different production seasons, and (iii) MFC-supporting microbes should be identified and retained to sustain MFC performance. This includes maintaining a high composition of electrogenic bacteria (i.e., *Clostridium* (OTU00515)), sulfide-oxidizing bacteria (i.e., *Hydrogenophilaceae* (OTU00713 and OTU01182), *Candidatus Chlorothrix* (OTU01036)), and methanotrophs (i.e., *Methylocaldum* (OTU00318 and OTU00322), *Candidatus Methylospira* (OTU00717)), while limiting the activity of sulfate-reducing bacteria (i.e., *Acinetobacter* (OTU00138)), and methanogens (i.e., *Methanolinea* and *Methanosaeta*) throughout the operation. Understanding the relationship between MFC performance and microbial activity helps in identifying the essential operating adjustments to keep the system optimal.

## Supporting information

S1 Table Number of raw reads and reads classified into OTUs. (XLSX)

S2 Table Microbial read counts and classification at the OTU level. (XLSX)

S3 Table Alpha-diversity indices for microbial communities in the MFC systems. (XLSX)

S4 Table Microbial classification into five groups: EB, SOB, SRB, MG, and MT, with references. (XLSX)

S5 Table OTUs exhibiting significantly higher abundance in M1L and inconsistency with M2L and M2H at the anode and reactor levels, using Spearman’s rank correlation |*ρ*| < 0.8. (XLSX)

S1 Fig Differential OTU abundance of anode-attached microbes and reactor microbes between M1L and M2L reactors. For anode-attached microbes: **A**, Volcano plot demonstrating significantly different OTUs between M1L and M2L using the criteria: a log-transformed FDR < 0.05 and an absolute log2 fold-change ≥ 2. 4 OTUs are downregulated while 42 OTUs are upregulated in M1L/M2L. **B**, Bar chart represents the fold-change of 4 OTUs with decreased abundance in M1L. **C**, The 42 OTUs show increased abundance in M1L when compared with M2L. For both anode-attached and suspension microbes: **D**, Volcano plot demonstrating significantly different OTUs between M1L and M2L using the criteria: a log-transformed FDR < 0.05 and an absolute log2 fold-change ≥ 2. 7 OTUs are downregulated while 38 OTUs are upregulated in M1L/M2L. **E**, Bar chart represents the fold-change of 7 OTUs with decreased abundance in M1L. **F**, The 38 OTUs show increased abundance in M1L when compared with M2L. Abbreviations in parentheses refer to OTUs that are highly specific to different taxonomic ranks: kingdom (K), phylum (P), class (C), order (O), family (F), and genus (G).
(TIF)

S2 Fig Differential OTU abundance of anode-attached microbes in M2H and M2L reactors. **A**, Volcano plot demonstrating significantly different OTUs between M2H and M2L using the criteria: a log-transformed FDR < 0.05 and an absolute log2 fold-change ≥ 5. 17 OTUs are downregulated while 84 OTUs are upregulated in M2H/M2L. **B**, Bar chart represents the fold-change of 17 OTUs with decreased abundance in M2H. **C**, The other 84 OTUs show increased abundance in M2H when compared with M2L. Abbreviations in parentheses refer to OTUs that are highly specific to different taxonomic ranks: kingdom (K), phylum (P), class (C), order (O), family (F), and genus (G).
(TIF)

S3 Fig Differential OTU abundance of anode-attached microbes and reactor microbes between M1L and M2H reactors. **A**, Volcano plot demonstrating significantly different OTUs between M1L and M2H using the criteria: a log-transformed FDR < 0.05 and an absolute log_2_ fold-change ≥ 5. 74 OTUs are downregulated while 43 OTUs are upregulated in M1L/M2H. **B**, Bar chart represents the fold-change of 43 OTUs with increased abundance in M1L. **C**, The other 74 OTUs show decreased abundance in M1L when compared with M2H. For both anode-attached and suspension microbes **D**, Volcano plot demonstrating significantly different OTUs between M1L and M2H using the criteria: a log-transformed FDR < 0.05 and an absolute log_2_ fold-change ≥ 5. 22 OTUs are downregulated while 30 OTUs are upregulated in M1L/M2H. **E**, Bar chart represents the fold-change of 30 OTUs with increased abundance in M1L. **F**, The other 22 OTUs show decreased abundance in M1L when compared with M2H. Abbreviations in parentheses refer to OTUs that are highly specific to different taxonomic ranks: kingdom (K), phylum (P), class (C), order (O), family (F), and genus (G).
(TIF)
