# Supplementary figures and images for: Optimization of microbial fuel cell performance application to high sulfide industrial wastewater treatment by modulating microbial function
Source: PLoS One. 2024 Jun 18;19(6):e0305673. doi: 10.1371/journal.pone.0305673 (PMC11185453; doi:10.1371/journal.pone.0305673)

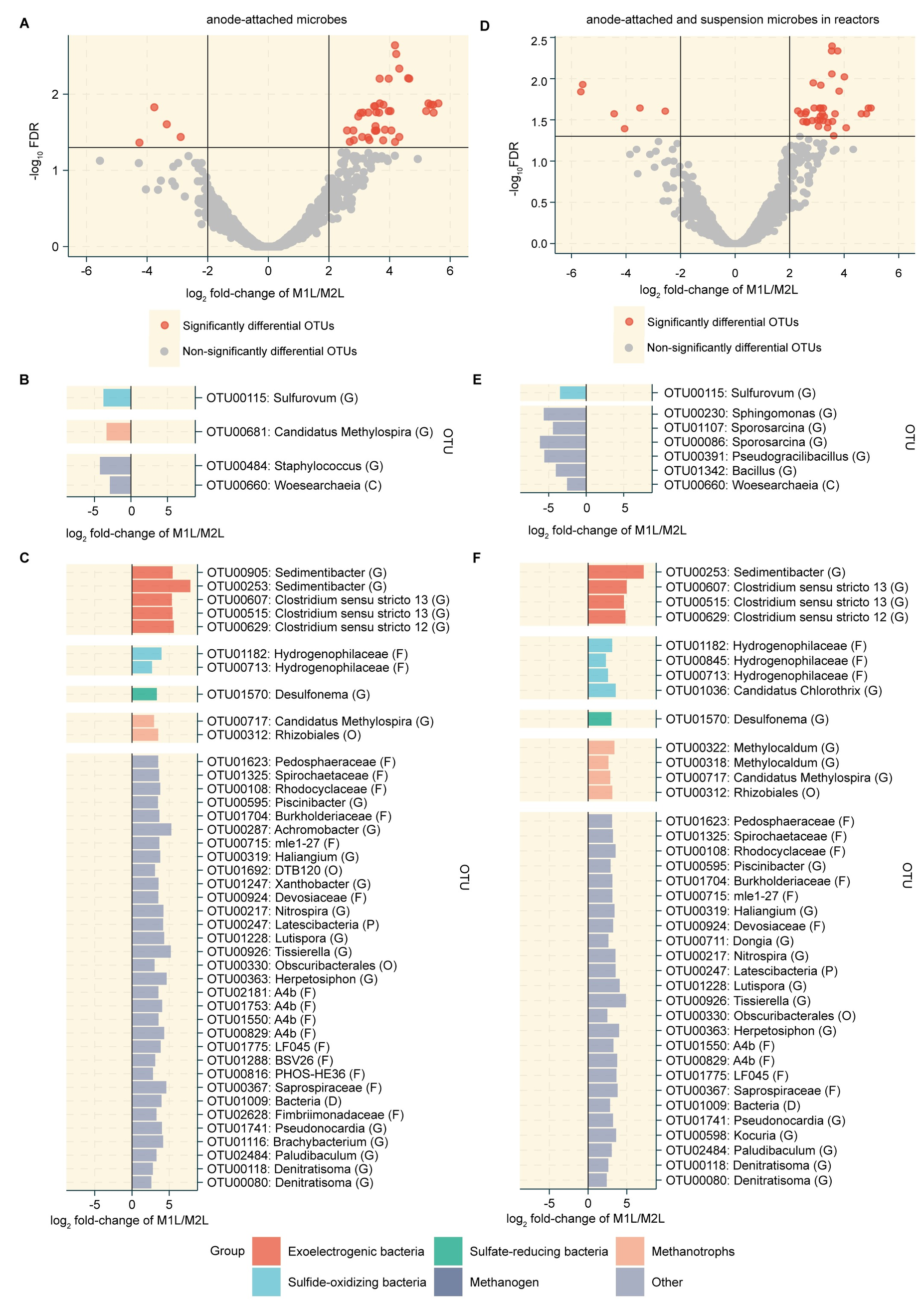

Supplement: S1 Fig — For anode-attached microbes: A, Volcano plot demonstrating significantly different OTUs between M1L and M2L using the criteria: a log-transformed FDR < 0.05 and an absolute log2 fold-change ≥ 2. 4 OTUs are downregulated while 42 OTUs are upregulated in M1L/M2L. B, Bar chart represents the fold-change of 4 OTUs with decreased abundance in M1L. C, The 42 OTUs show increased abundance in M1L when compared with M2L. For both anode-attached and suspension microbes: D, Volcano plot demonstrating significantly different OTUs between M1L and M2L using the criteria: a log-transformed FDR < 0.05 and an absolute log2 fold-change ≥ 2. 7 OTUs are downregulated while 38 OTUs are upregulated in M1L/M2L. E, Bar chart represents the fold-change of 7 OTUs with decreased abundance in M1L. F, The 38 OTUs show increased abundance in M1L when compared with M2L. Abbreviations in parentheses refer to OTUs that are highly specific to different taxonomic ranks: kingdom (K), phylum (P), class (C), order (O), family (F), and genus (G). (TIF) [file pone.0305673.s006.tif]

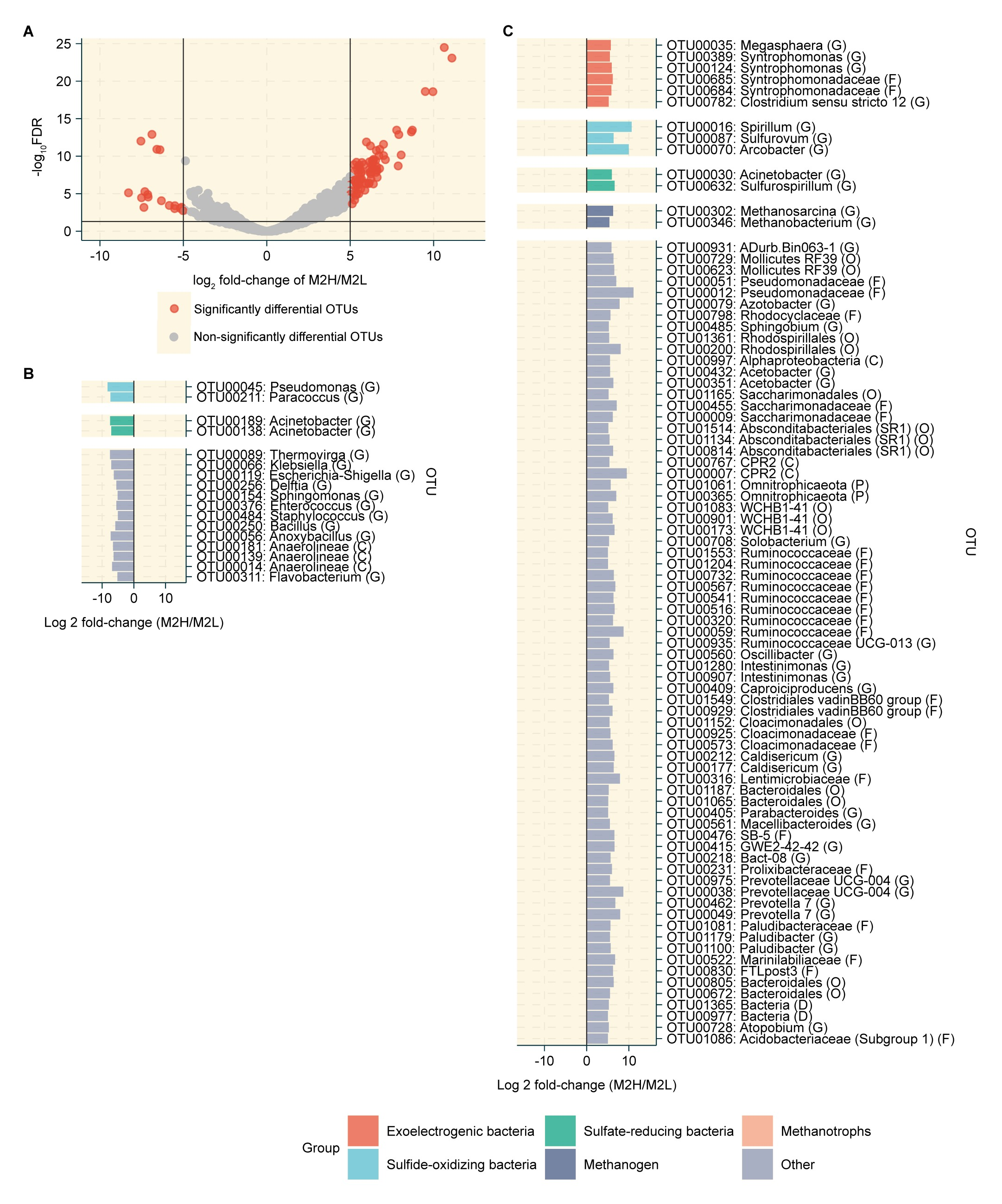

Supplement: S2 Fig — A, Volcano plot demonstrating significantly different OTUs between M2H and M2L using the criteria: a log-transformed FDR < 0.05 and an absolute log2 fold-change ≥ 5. 17 OTUs are downregulated while 84 OTUs are upregulated in M2H/M2L. B, Bar chart represents the fold-change of 17 OTUs with decreased abundance in M2H. C, The other 84 OTUs show increased abundance in M2H when compared with M2L. Abbreviations in parentheses refer to OTUs that are highly specific to different taxonomic ranks: kingdom (K), phylum (P), class (C), order (O), family (F), and genus (G). (TIF) [file pone.0305673.s007.tif]

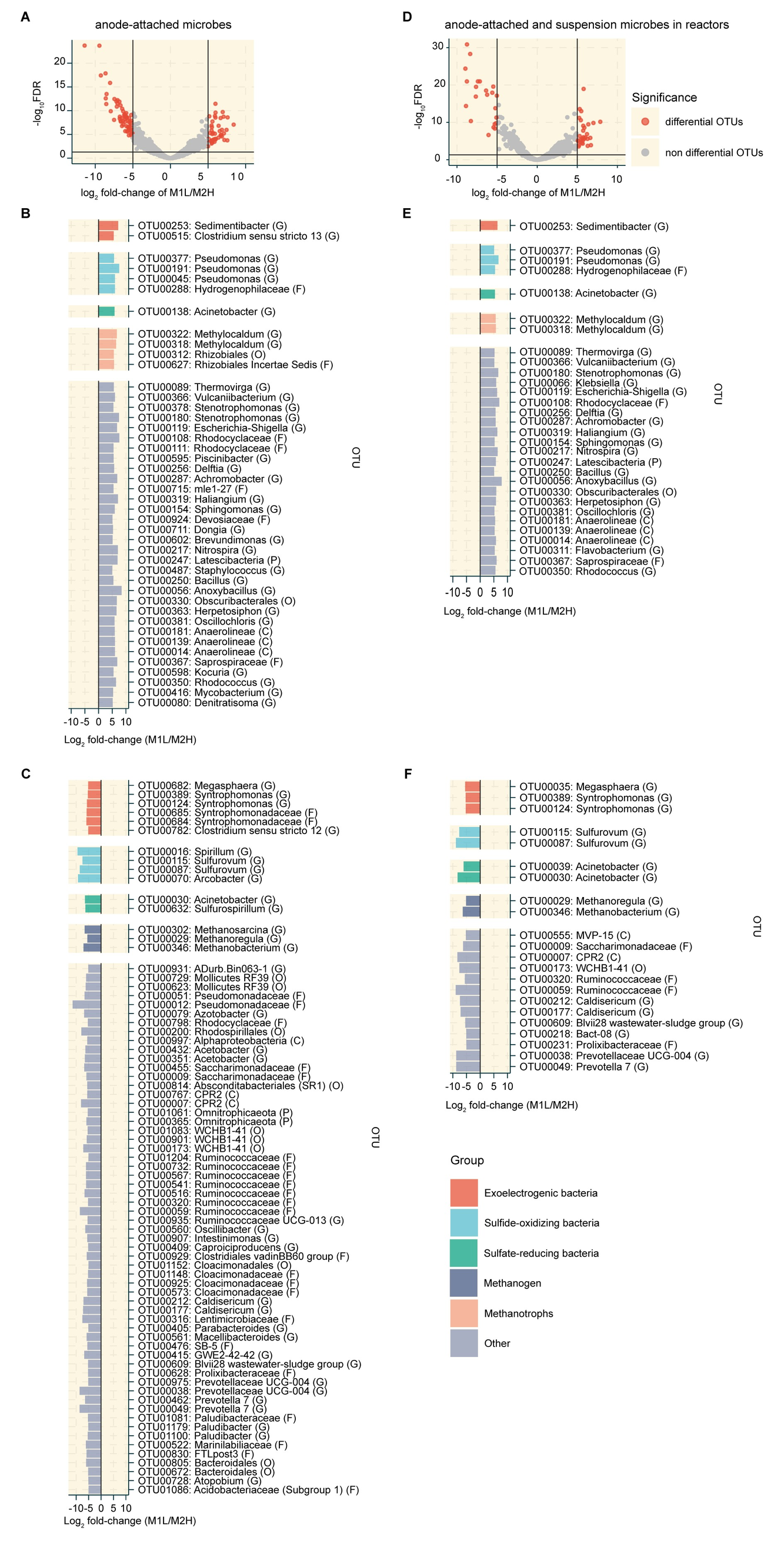

Supplement: S3 Fig — A, Volcano plot demonstrating significantly different OTUs between M1L and M2H using the criteria: a log-transformed FDR < 0.05 and an absolute log2 fold-change ≥ 5. 74 OTUs are downregulated while 43 OTUs are upregulated in M1L/M2H. B, Bar chart represents the fold-change of 43 OTUs with increased abundance in M1L. C, The other 74 OTUs show decreased abundance in M1L when compared with M2H. For both anode-attached and suspension microbes D, Volcano plot demonstrating significantly different OTUs between M1L and M2H using the criteria: a log-transformed FDR < 0.05 and an absolute log2 fold-change ≥ 5. 22 OTUs are downregulated while 30 OTUs are upregulated in M1L/M2H. E, Bar chart represents the fold-change of 30 OTUs with increased abundance in M1L. F, The other 22 OTUs show decreased abundance in M1L when compared with M2H. Abbreviations in parentheses refer to OTUs that are highly specific to different taxonomic ranks: kingdom (K), phylum (P), class (C), order (O), family (F), and genus (G). (TIF) [file pone.0305673.s008.tif]
